# The Implementation of Evidence-Based Psychiatric Rehabilitation: Challenges and Opportunities for Mental Health Services

**DOI:** 10.3389/fpsyt.2019.00147

**Published:** 2019-03-20

**Authors:** Antonio Vita, Stefano Barlati

**Affiliations:** ^1^Department of Mental Health and Addiction Services, ASST Spedali Civili, Brescia, Italy; ^2^Department of Clinical and Experimental Sciences, University of Brescia, Brescia, Italy

**Keywords:** severe mental illness, psychosocial rehabilitation, evidence-based practice, recovery, person-centered treatment

## Background

In the recent past, mental health care and psychiatric service organization conceptually and structurally changed. The introduction of antipsychotic drugs in the 1950s substantially changed the treatment of schizophrenia and other psychotic disorders (1). The consequent deinstitutionalization decreased the number of hospitalizations and transferred the treatment pattern from an inpatient care to community-based outpatient services, although the latter has grown up differently through and within the countries (2).

In recent years, treatment of severe mental illness (SMI) shifted from management and stabilization of symptoms, to the much broader and more ambitious goal of achieving functional recovery. Despite advances in pharmacological treatment of people with SMI, it has become clear that medications alone are not sufficient to achieve a full symptom remission and functional recovery (3–5).

The effectiveness of drug treatments is further impaired by the total or partial non-adherence, affecting more than half of patients with SMI (6, 7). In this context, several non-pharmacological interventions have been developed for SMI and, among them, psychosocial rehabilitation represents one of the most relevant systematic effort to help adults with psychiatric disabilities to achieve their personal goals.

## Psychiatric Rehabilitation: the Bases of Evidence

Psychiatric rehabilitation has a bidirectional focus, seeking to influence both the individuals' strengths and challenges related to these goals, and the community contexts in which the persons will live them out (8, 9). The World Psychiatric Association (WPA) highlighted that the aim of psychosocial rehabilitation is to support people with SMI in developing their cognitive, emotional and social skills, in order to live in the community with the slightest professional sustenance. The premise of psychosocial rehabilitation is that, beyond clinical severity, each patient has strengths and resources on which rehabilitation could be addressed (10). For a long time and in different contexts, psychiatric rehabilitation was considered a second or a third line treatment, to be applied only when other types of intervention had failed. Moreover, too often psychosocial rehabilitation has been considered a therapeutic practice of “common sense,” which could be carried out by any mental health professional, even without specific training. Lastly it was mostly considered a way to spend and fill the day, rather than a real specialized treatment with a specific aim (8).

In recent years, however, psychiatric rehabilitation has been better defined and its starting assumptions and paradigms have been refined and consolidated by evidence-based research. Now psychiatric rehabilitation offers structured interventions, defined by approved procedures and accurate assessment tools and measures, with clear target, in order to achieve specific outcomes in patients with SMI (11).

In the last few years, several research groups in different countries identified a wide and increasing body of effective and effectiveness psychosocial rehabilitation practices (9, 12, 13). Evidence-based psychiatric rehabilitation models have been developed for numerous objectives, including employment, independent living and community living skills (14–16). Evidence-based practices (EBPs) for SMI include, among others, Assertive Community Treatment (ACT), cognitive-behavioral therapy for psychosis (CBTp), cognitive rehabilitation, family psychoeducation, illness self-management training, social skills training (SST), and supported employment (11, 17). Table 1 summarizes the current evidence-based psychiatric rehabilitation interventions and their potential benefits in SMI (11, 12, 17–25).

**Table 1:** Evidence-based psychiatric rehabilitation interventions for SMI.

**Evidence-based intervention**	**Main outcomes**
Assertive community treatment	Decrease in length hospitalization and homelessness rates
Illness self-management training	Skills improvement to cope with the illness, relapses reduction and social functioning improvement
Cognitive behavioral therapy for psychosis	Positive and negative symptoms reduction, mood and social functioning improvement
Family interventions/psychoeducation	Relapses reduction, social functioning improvement, increase in treatment adherence, illness knowledge, family coping and decrease in family burden
Social skills training	Negative symptoms reduction, social skills and social functioning improvement
Cognitive remediation, including social cognitive and metacognitive training	Negative symptoms reduction, cognitive, social cognitive, metacognitive and psychosocial functioning improvement
Supported employment	Improvement in employment rates, hours worked and QoL
Physical aerobic exercise, including healthy lifestyle intervention	Positive and negative symptoms reduction, mood, cognition, QoL and social functioning improvement
Integrated early intervention for psychosis	Positive and negative symptoms reduction, treatment adherence, QoL, and social functioning improvement
Integrated intervention for comorbidity with SUD	Decrease in substance use and detention, improvement in social functioning
Psychoeducation for bipolar disorder	Decrease in illness recurrence, length and rates of hospitalization, increase in illness knowledge and treatment adherence, decrease in caregiver burden
Functional remediation for bipolar disorder	Cognitive and psychosocial functioning improvement
Dialectical behavior therapy—skills training groups—for BPD	Reduction in anger, suicidal and self-injurious behaviors

*BPD, borderline personality disorder; QoL, quality of life; SMI, severe mental illness; SUD, substance use disorder*.

The term EBP takes into account those interventions shown to be effective in improving illness course and outcome, based on rigorous studies methodologically well-designed and conducted (26). There is a consensus that EBPs need standardized interventions, with the aim to improve symptoms and functioning in people with SMI. Furthermore, EBP must be supported by a number of randomized controlled trials (RCTs), at least two of them conducted by different research groups (27).

On the other hand, other psychosocial interventions are diffused and widely applied, although not yet evidence-based. Despite few studies or reviews are now available, some data have shown that additional use of the so-called expressive therapies, in particular art therapies, seems to reduce negative symptoms among schizophrenia patients, being able to reach an improvement in self-knowledge, awareness, as well as affective and relationship skills in this population (28–30). Moreover, two core concepts of psychosocial rehabilitation have emerged: (i) all resources and professionals involved in the rehabilitative process should be coordinated and integrated with the aim to optimize treatment, and (ii) the same therapeutic team should assure the continuity not only in various contexts of care, but also in subject's life context (31).

## Evidence-Based Psychiatric Rehabilitation: Critical Issues and Future Challenges

Currently, the two major challenges of mental health in increasing the quality of psychiatric services are: (i) redesigning services on the principles of recovery and (ii) implementing services that deliver interventions supported by scientific evidence. The two strategies are complementary and should be integrated with each other. At present, there is the risk of not of neither a proven effective intervention, nor a really person-centered treatment approach. Despite the growing scientific literature on this topic, the most serious problem is that evidence-based rehabilitative interventions are not widely available in real-world practice. The science-to-service gap—that is, the gap between practices knowledge effective and that available and provided in mental health services—is one of the most relevant problems in the public mental health system (32).

Although evidence-based psychosocial interventions led to clearly promising findings, there are still some doubts and uncertainties regarding their usefulness and feasibility in the daily clinical practice of mental health services. For many years, high-income European countries continued to invest funds and resources in old and too expensive not-evidence-based, not-recovery-oriented and not-personalized care services. The major investments still concern psychiatric acute care, hospitalization and residency, day centers and resocialization activities carried out within psychiatric services (33). Even today, only a few patients with SMI receive a suitable evidence-based psychosocial rehabilitation treatment (34, 35). One of the crucial obstacles in developing of an evidence-based and recovery-oriented community mental health service is that, not only patients and professionals have different views about mental illness, but also among the same professionals coexist different beliefs on this field, which prevent the realization of a harmonic project designed and tailored on the real patient needs. In addition, there also are divergences on treatment goals: physicians and professionals often emphasize clinical stability and symptom control as primary goals, whereas people with SMI feel essential achieving improvements of their psychosocial functioning and life satisfaction (36).

Another weakness in that psychiatric rehabilitation interventions lack of enough evidence about their precise indications, predictors of response and the presence of any contraindications and/or adverse effects. Not least, it should be taken into careful consideration the clinical studies methodology, such as the choice of outcome measures and the presence of confounding factors, which may give rise to different and/or wrong interpretations (1). Evidence-based, person-centered, recovery-oriented psychosocial rehabilitation could provide the assumptions and the theoretical premises necessary for the development of a shared community-based care system. The biggest challenge of modern psychiatry, in implementing evidence-based rehabilitation services, is not the lack of resources themselves, but rather the lack of efficient skills at the organizational level, able to utilize and allocate the resources according to a clear understanding of the real needs of a mental health service (33).

The situation is not so different in the USA; although the USA was among the first countries in the world to address the issue of evidence-based psychiatric rehabilitation services implementation, the quality of rehabilitative services offered in the USA seems to be even behind the other high-income countries (37).

Moreover, mental health costs comprehend consistent funding for hospital and residential treatment and psychotropic drugs, significantly unbalanced with the funding allocated for psychosocial, recovery-oriented interventions (37).

## Evidence-Based Psychiatric Rehabilitation: Implementation Strategy

It is now important to emphasize how the implementation of evidence-based and recovery-oriented interventions takes a long time to be acquired by mental health workers and to overcome their resistance to change. Moreover, the achievement of these objectives also requires a clear leadership direction and a constant commitment to teach the staff the new techniques and to learn from their experimentation. Leadership should be strongly committed to providing resources and supports, giving relevance to continuing education on work-place, developing quality measures and recovery indicators that include the use of new practices. Furthermore, leadership should have the courage to eliminate inefficient practices often defended by mental health worker, families, politicians and other stakeholders. It often happens that the new learned techniques are neither transmitted to the working group and are nor they applied in the real-world setting.

In this context, the public resources invested in staff training remain unused assets of a few professionals and are far from the everyday clinical practice. Research in the field of EBP demonstrated that the learning of theory alone does not significantly influence professional behavior and that the introduction of innovative treatments in the real practice occurs only if it responds to the mental health workers specific needs. According to Williamson et al. (38) there is a need of different dissemination strategies (training events, written materials, practical guidelines that support clinical decision-making) and reinforcement strategies (provide feedback on interventions and same time remember theory) to increase the skills. In particular, motivational interventions should teach the skills necessary to change, also supporting the elimination of the barriers that delay the EBP implementation process. For these reasons, several papers on EBP implementation in health care with the aim of transferring theoretical assumptions into practice were published over the last years, but only few guidelines have been defined in this field (39).

Implementing EBP and recovery in mental health are complex processes that require not only a real transformation of paradigm and working modalities, but also a cultural change, updating the knowledge and information baggage applied for years. The latter represents one of the greatest obstacles and one of the most deep-rooted resistances to change in a mental health service. It is therefore not enough that just a few learn the skills, but rather the entire working group, or better the whole care system, should acquire all the strategies and tools that allow homogeneous levels of performance, with the purpose to apply the new methods and techniques learned and to assess their impact at all the mental health services levels.

## Conclusion and Future Direction

The existing literature on psychiatric rehabilitation delivery is still scarce all over the world. In particular, the operational translation of the psychiatric rehabilitation evidences and its theoretical concepts has never been the object of a careful analysis in order to verify which rehabilitation activities and techniques are actually offered in the mental health delivery system.

Current psychosocial rehabilitation practices are highly variable in terms of methodology and contents, with relevant differences from one country to another, and also within the same country, according to the specific orientation and tradition characterizing each Department of Mental Health.

There is the need to increase the knowledge and awareness about the state of the art of different systems of management and funding of psychosocial rehabilitation in the “real-world” settings. It is also crucial to reveal commonalities and divergences as well as strengths and limits of the various assets in the different countries. Furthermore, specific pathways should be designed and implemented with the aim to overcome all the personal, social, organizational and political barriers that deny evidence-based treatments for patients and their families.

Moreover, it is now pivotal to increase our knowledge on: (i) different types of interventions, (ii) modalities of delivery of interventions, such as intensity and duration of treatments, (iii) assessment tools, (iv) outcome measures, (v) predictors of treatment response, (vi) persistence of intervention efficacy, and (vii) integration with pharmacological therapies and with other psychosocial interventions. The new acquired knowledge should be framed inside a personalized intervention, also taking into account patient's preference.

In this context, scientific societies in different countries could play an important role in defining the best strategies to promote and disseminate among the stakeholders the principles underlying a patient-centered and evidence-based psychosocial rehabilitation. The same goal could also be achieved by sharing and disseminating scientific research in the field of psychosocial rehabilitation. In this regard, the Social Psychiatry and Psychiatric Rehabilitation section in the *Frontiers in Psychiatry* journal aims at publishing original studies that advance the evidence and understanding of psychosocial treatment and recovery of people with SMI (such as schizophrenia spectrum disorders, mood disorders, personality disorders, eating disorders, and others), consistent with the principles and values of psychiatric rehabilitation and person-centered care. Frontiers in Psychiatry—Social Psychiatry and Psychiatric Rehabilitation will describe the psychiatric rehabilitation process and aims to differentiate psychosocial interventions that can be classified as evidence-based psychiatric interventions from other not or not yet evidence-based. The new Frontiers section will examine the main psychiatric rehabilitation interventions within the framework of the psychiatric rehabilitation process, taking into account the evidence of their efficacy, effectiveness and efficiency. Psychiatric rehabilitation interventions are currently a mixture of evidence-based practices, promising practices and emerging methods that can be effectively tied together, providing a broad strategy to achieve personal functional recovery.

## Author Contributions

AV and SB participated in the writing process of the first draft of the manuscript, revised and approved the final version of the manuscript.

### Conflict of Interest Statement

The authors declare that the research was conducted in the absence of any commercial or financial relationships that could be construed as a potential conflict of interest.
